# Role of B-cell receptors for B-cell development and antigen-induced differentiation

**DOI:** 10.12688/f1000research.13567.1

**Published:** 2018-04-06

**Authors:** Juan Carlos Yam-Puc, Lingling Zhang, Yang Zhang, Kai-Michael Toellner

**Affiliations:** 1 Institute of Immunology and Immunotherapy, College of Medical and Dental Sciences, University of Birmingham, Birmingham, UK

**Keywords:** B cell receptor, B cell differentiation, B cell development

## Abstract

B-cell development is characterized by a number of tightly regulated selection processes. Signals through the B-cell receptor (BCR) guide and are required for B-cell maturation, survival, and fate decision. Here, we review the role of the BCR during B-cell development, leading to the emergence of B1, marginal zone, and peripheral follicular B cells. Furthermore, we discuss BCR-derived signals on activated B cells that lead to germinal center and plasma cell differentiation.

## Introduction

B cells, the antibody-producing cells, have a protagonist role in the immune response. Although the presence and relevance of antibodies were established more than 100 years ago ^1^ and antibody-producing cells were identified in the mid-20th century ^2^, it took until 1965 for the distinctive B-cell lineage to be recognized ^3, 4^. Today, there are still many questions about B-cell differentiation during development and after activation and about the signals that govern such differentiation.

B cells undergo a diversification process during their development in bone marrow and fetal liver, and, as part of this differentiation, B cells rearrange the immunoglobulin (Ig) heavy (H) and light (L) chain gene loci to create a complete Ig molecule ^5, 6^. The commitment of the common lymphoid progenitor to the B-cell lineage can be recognized by the expression of the B220 isoform of CD45 ^6– 8^. B cells then develop through several well-characterized stages, ending with the expression of surface IgM and IgD class Ig molecules, which, in association with Igα and Igβ, form the B-cell receptor (BCR) for the antigen ^5, 6^. BCR signaling is required for B-cell maturation and survival, and BCR must provide tonic signals, either spontaneously or on interaction with ligands in the environment ^9– 12^. After this, B cells emerge to recirculate through secondary lymphoid organs such as the spleen and lymph nodes ^13^. Being well positioned in the secondary lymphoid organs, mature naïve B cells are ready to respond to antigens. Recognizing the antigen through the BCR, B cells are activated and differentiate into plasma cells through extra-follicular differentiation ^14^, or they become germinal center (GC) precursor cells to start GC reactions ^15, 16^. The precise signaling mechanisms of B-cell fate decision during this stage are not entirely understood.

**Figure 1.**
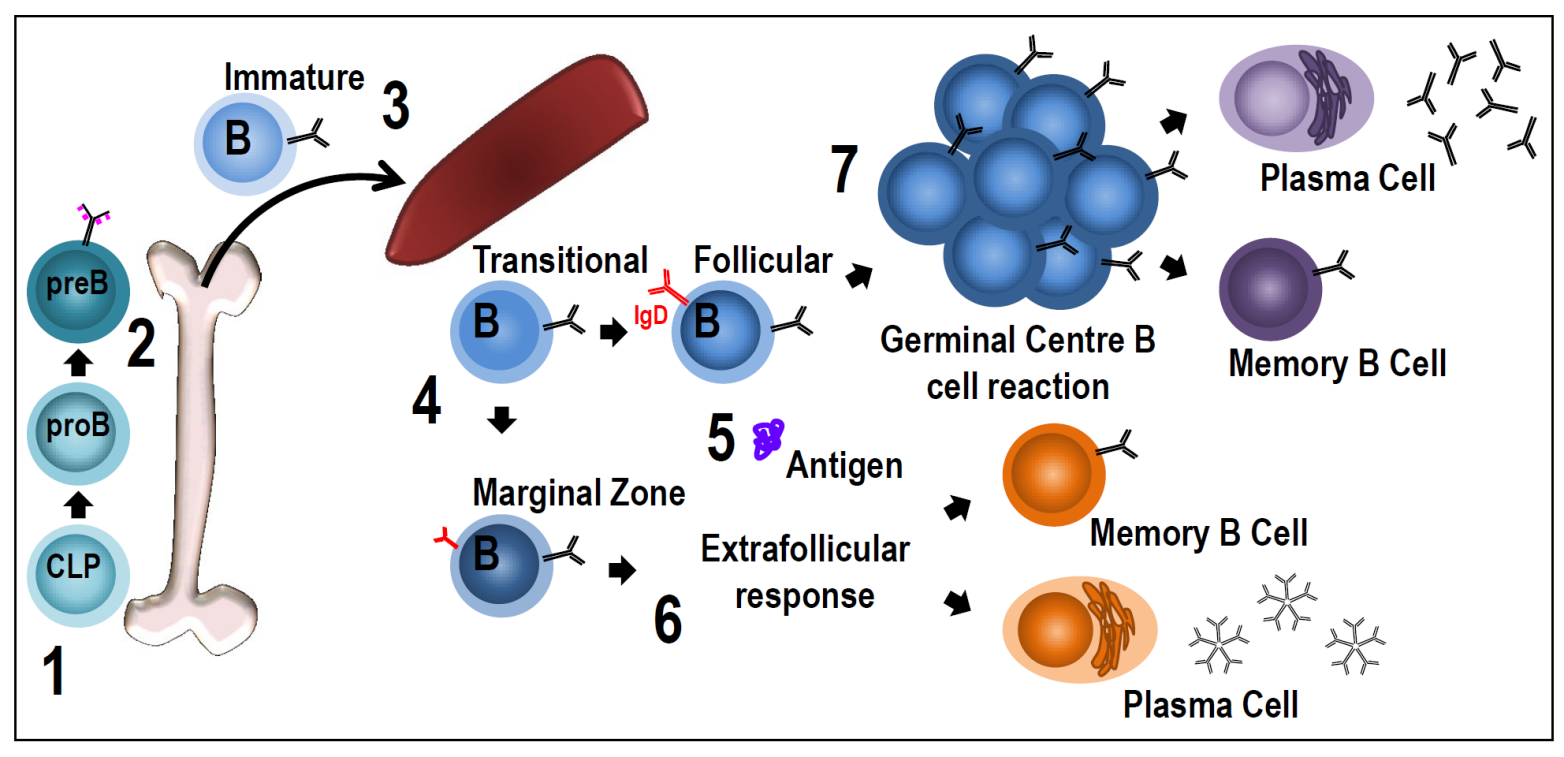
B-cell receptor signaling during B-cell development and for B-cell differentiation after the encounter with the antigen. **(1)** Common lymphoid progenitor (CLP) cells commit to the B-cell lineage when they start expressing the B220 isoform of CD45. **(2)** Pro-B cells undergo DJ rearrangements and become pre-B cells when they express membrane forms of μ heavy chains with surrogate light chains (pink dotted lines) in the pre-B receptor. The transition between pro-B and pre-B cells has been described as being dependent on membrane association of Igα/Igβ complexes and their ability to generate basal signals. It seems that pre-B receptors do not have a ligand-recognition function ^40, 41^. **(3)** After VDJ recombination in the pre-B cell stage, immature B cells pair light chains with μ chains to form monomeric IgM, which is expressed at the cell surface in association with Igα/Igβ to form the B-cell receptor (BCR). Newly formed immature B cells exit the bone marrow to reach the spleen, where, as transitional B cells, they will complete their maturation before entering the follicles or the marginal zone (MZ). **(4)** BCR signaling strength appears to have a critical role during follicular (Fo) or MZ B-cell differentiation. There is evidence of at least two possibilities ^29, 30, 36, 42– 44^: increased BCR signaling could drive differentiation to Fo B cells or to MZ B cells. This could depend on other factors such as the microenvironment or the timing of BCR signaling. **(5)** Mature naïve B cells are ready to respond to antigens. By specifically binding antigen through the BCR, B cells are activated and differentiate into plasma cells through extra-follicular differentiation **(6)** or start the T-cell-dependent germinal center (GC) reaction **(7)**. The mechanism of activated B cells entering the GC reaction or undergoing rapid plasma cell differentiation in extra-follicular proliferative foci is controlled by the nature of the interaction between the BCR and antigen. Responding clones that undergo a strong initial interaction with antigen can efficiently differentiate into extra-follicular plasma cells ^45^. However, it has also been shown that B cells expressing higher-affinity BCRs are more competitive to become pre-GC B cells during T–B interaction ^46, 47^. This seems contradictory, but timing may be a very important factor. B cells in the GC reaction are selected on the basis of their interaction with the antigen through BCRs. Based on how efficiently B cells present antigen to Fo T helper cells, they are allowed to further differentiate inside the GC. Whether this BCR–antigen interaction results in significant signaling and has a role for selection is not clear.

In this review, we will focus on B-cell subsets in the spleen, key steps of differentiation after activation, and, in particular, recent findings about the role of the BCR driving these distinct differentiation stages ( Figure 1).

## Brief overview of B-cell receptor signaling

BCR signaling has been intensely studied over the last 30 years, and details of this complex process are still not fully understood. In-depth reviews have been produced by others ^17– 20^. In brief, BCR is activated by binding of the antigen. This leads to phosphorylation of immunoreceptor tyrosine-based activation motif (ITAM) by the first kinase in the BCR signaling pathway, primarily LYN (part of the *src*-kinase family). After this, SYK is recruited through its SH2 domain to the phosphorylated Igα–Igβ heterodimer. The higher propensity of ligand-bound BCR molecules to aggregate can enhance their association with *src*-family PTKs ^17, 20^. Once SYK is activated, the BCR signal is propagated via a group of proteins associated with the adaptor protein B-cell linker (BLNK, SLP-65). Phospho-BLNK serves as a scaffold for the assembly of the other components, including Bruton’s tyrosine kinase (BTK), VAV 1, and phospholipase C-gamma 2 (PLCγ2) ^19^. The initiation of the BCR signal is indirectly regulated by at least two non-receptor-associated molecules: B220 and C-terminal *src* tyrosine kinase (CSK) ^17^.

Additionally, following BCR ligation, tyrosines of the cytoplasmic tail of CD19 are phosphorylated by LYN to create binding sites for the SH2 domains of the p85 adaptor subunit of PI-3K as well as other SH2 domain-containing effectors. Activated PLCγ2 cleaves membrane-associated phosphoinositide PI(4,5)P2 into the second messengers I(1,4,5)P3 and DAG. I(1,4,5)P3 generation causes the mobilization of Ca ^2+^ from intracellular and extracellular stores. Ca ^2+^ signaling is required for the activation of transcription factors such as nuclear factor kappa B (NF-κB) and nuclear factor of activated T cells (N-FAT) by protein kinase C (PKC) ^21^. DAG represents a classic activator of PKC isotypes which regulate the mitogen-activated protein kinase (MAPK) family (extracellular signal-regulated kinase [ERK], c-Jun NH2-terminal kinase [JNK/SAPK], and p38 MAPK); the overall result of these processes drives activation of the B cell, antigen presentation, cytokine production, and cell proliferation and differentiation ^17– 19^. In the following, we will discuss the effects of BCR signaling during B-cell development and after the encounter with the antigen.

## B1 B cells

Three major populations of mature B cells have been described in mice: B1, marginal zone (MZ), and follicular (Fo) B cells. The phenotypic, microanatomic localization and functional differences that characterize them suggest specialized functions linked to the niches in which they reside ^5^. B1 cells produce polyreactive natural antibodies (NAbs) of relatively low affinity and primarily of the IgM isotype ^22^. NAbs play a critical role in the innate immune response against pathogens, and their absence can lead to increased susceptibility to microbial infections ^23– 25^. B1 cells are the major subpopulation in pleural and peritoneal cavities; however, they can also be found in the spleen and other lymphoid organs but at low frequency ^26^. B1 cells consist of two functional specialized subpopulations regarding the expression of CD5: CD5 ^+^ B1a and CD5 ^−^ B1b cells ^27^. However, expression of Blimp-1 also delineates two main coexisting B1 subpopulations in the bone marrow and spleen: B1 Blimp-1 ^hi^ cells that are dependent on Blimp-1 for maximal natural Ig production and those B1 cells that neither express Blimp-1 nor require it for steady-state antibody production ^28^. Recently, it has been shown that interleukin 17A (IL-17A) promotes B1-cell infiltration into lungs during viral infection, where B1a cells differentiate into IgM-producing plasma cells. This process was promoted through Blimp-1 expression and NF-κB activation ^25^. It is conceivable that the regulation of Blimp-1 expression would also drive the functional role of B1 subsets in sites such as the lung.

What is the role of BCR signaling in B1-cell differentiation? Studies with genetically modified mice indicate that the strength of BCR signaling may control the development or persistence of B cells (or both) ^29– 36^. Mutations that enhance BCR signaling strength through the specific deletion of SHP-1 in B cells expand the B1a population. SHP-1 is a protein-tyrosine phosphatase expressed in hematopoietic cells and plays a signal-attenuating role in pathways initiated by many ITAM-containing receptors ^37– 39^. The signal-attenuating effects of SHP-1 are mediated primarily via its binding to inhibitory receptors, such as CD5, expressed by B1a cells ^34^. Additionally, enhanced tonic BCR signaling results in an increased B1 B-cell subpopulation and a dysregulated homeostasis of other B-cell subsets ^33^. These findings indicate that BCR signaling is important in fate decisions during B1 cell development, and further studies are needed to better understand these mechanisms.

## Marginal zone B cells

MZ B cells contribute about 5–10% of the B cells in the spleen. The “marginal zone” designation refers to the splenic structure that separates the red and the white pulp adjacent to the marginal sinus, where—in both mice and humans—these MZ B cells are in direct contact with blood and its contents ^5, 48^. The specialized localization and migration of B cells are strictly regulated under the guidance of different chemokine–chemokine receptor pairs, such as CXCL13–CXCR5, S1PR1, and S1P ^49– 53^.

Blood from the primary sinusoids in the spleen perfuses the MZ; the anatomic location of MZ B cells facilitates their role as a rapid first line of defense against blood-borne particulate antigens ^52, 54^. After MZ B cells capture the antigen, they transport it to the follicles and deliver it to follicular dendritic cells (FDCs) ^52, 53, 55^. Furthermore, MZ B cells respond to thymus-independent type 2 antigens producing high quantities of IgM and IgG3 ^14, 56, 57^.

Newly formed B cells exiting the bone marrow reach the spleen at a relatively immature stage; these are termed transitional B cells and they need to complete their maturation in the spleen before entering the follicles or the MZ ^58^. It has been described that B cells in the transitional 2 (T2) stage face a decision to mature into either Fo or MZ B cells ^5, 48^. However, very recently, it was shown that T1 B cells can differentiate to MZ B cells ^32^. During this differentiation, signaling through the BCR is important for the Taok3-mediated acquisition of membrane expression of ADAM10, which cleaves Notch2 and CD23 ^31, 32^. MZ B-cell instruction requires triggering of Notch2 on developing B cells by Delta-like 1 (Dll1) expressed by splenic red pulp sinus endothelial cells or MZ reticular cells ^35, 59^. How exactly B-cell-positive selection and BCR signaling are causing Taok3 activation and ADAM10 surface expression will require further study ^32^. Furthermore, BCR signaling strength appears to have a critical role during MZ B-cell development; mice lacking secreted IgM displaying increased BCR signaling had increased MZ and decreased Fo B-cell numbers ^36, 42, 43^. However, some studies reported that MZ B cells need low BCR signaling strength for their differentiation but that transitional B cells with higher BCR signaling strength favor differentiation into Fo B cells ^29, 30, 44^.

## Follicular B cells

Fo B cells are the most prevalent of the three subsets of B cells and the better-studied subpopulation. Their anatomic enrichment in primary follicles gives them their name; however, they are not confined to the follicles and also predominate among the mature populations of B cells in the bone marrow, blood, and other lymphoid organs ^5^. Fo B cells are involved mainly in interactions with T cells, and their responses to T-cell-dependent antigens eventually originate GC reactions ^16^. Many of the mechanisms producing the selection of B-cell subsets, the roles of self- and environmental antigens, and survival signals that drive or maintain them in their proper anatomic and functional niches remain to be elucidated. BCR signaling has been proposed to be crucial in the selection of B1, MZ, and Fo B cells, supported by different genetically manipulated mice where the altered BCR signaling affected different B-cell subsets ^32– 34, 36^.

## Follicular B-cell activation

After Fo B cells encounter the antigen through the BCR, CXCL13–CXCR5 slows down the motility of B cells by promoting membrane ruffling and LFA-1-supported adhesion to facilitate the antigen-recognizing process and enhance B-cell activation ^60^. Meanwhile, surface expression levels of CCR7 on responding B cells increase rapidly to make them more sensitive to CCL19/CCL21 ^51^. CCL19/CCL21 is expressed by T-zone reticular cells and extends a gradient to the Fo region. Along the chemokine gradient, antigen-engaged B cells migrate from follicles to T–B border in order to get signals from primed T cells ^51, 61, 62^. EBI2 drives B cells to move back to the outer follicle and inter-follicular regions ^63, 64^. These results indicate interplay between activated BCR downstream signaling and surface chemokine receptors ^60, 61^. Although the possibility is less studied on steady state, tonic BCR signaling could also regulate chemokine receptor expression during their anatomic enrichment in specific areas within the secondary lymphoid organs.

## Germinal center B cells

After T–B cell interaction, activated B cells either differentiate into plasma cells in the extra-follicular response ^14^ or become GC precursor cells, migrating back into follicles to start GC reactions ^16^. To get co-stimulation from T cells, B cells need to present cognate antigens to T cells in the major histocompatibility complex II (MHC-II) context. B-cell-captured antigen goes through both extracellular and intracellular degradation to become peptides ^65^. These peptides then are assembled with MHC-II molecules and expressed on the B-cell surface as peptide–MHC (pMHC). pMHC–T-cell receptor recognition has an essential role in the process of T–B “pairing” to ensure the specificity of later reactions. Schwickert *et al*. showed that a higher amount of pMHC help activated B cells, locking T-cell help on the T–B border, thus enabling them to become GC precursor B cells ^46^. CD40L is the most important cognate signal delivered from T cells. In mice and humans, CD40–CD40L ligation is indispensable for the initial formation of GCs ^66, 67^ and is needed to maintain ongoing GC reactions ^68^.

BCR affinity also plays an important role during the initial GC B-cell fate decision. The mechanism of activated B cells entering the GC reaction or undergoing rapid plasma cell differentiation in extra-follicular proliferative foci is controlled by the nature of the interaction between the BCR and antigen. Responding clones that undergo a strong initial interaction with antigen can efficiently differentiate into extra-follicular plasma cells and contribute to the rapid early thymus-dependent ****antibody response ^45^. Although the requirements for GC entry are not stringent ^69^, responding B cells expressing higher-affinity BCRs on their surface are more competitive to become pre-GC B cells during their T–B interaction ^46, 47^. High-affinity BCR captures more soluble antigens ^70^ and leads to a higher amount of pMHC expressed on the B-cell surface, which results in a competitive cognate interaction with T cells. The durations of T–B interactions have critical roles in B-cell fate decisions. Recent research shows that ICAM-1/2 adhesion molecules on B cells can secure long-lasting T–B interactions to enhance T-cell help. The expression of ICAM-1/2 could compensate the lack of MHC-II signaling to form GC B cells ^71^.

BCRs of B cells differentiating in GCs have to interact with antigen repetitively. Whether the BCR-antigen interaction in the GC results in significant signaling and what the role of this is are under debate ^72– 74^. The interaction is certainly important to test BCR specificity and binding competitiveness, probably mainly in competition with antibodies present in immune complexes on the FDC networks ^75, 76^. More important than BCR signaling may be that the affinity of the BCR–antigen interaction will let B cells take up more or less antigen, resulting in more or less efficient positive selection by Fo T helper cells ^47, 74, 76^.

Much of the knowledge on B-cell differentiation in response to antigen has been gained by using BCR knock-in animals specific for haptens or single epitopes on model proteins ^46, 77^. Recent attempts to use complex protein antigens such as influenza hemagglutinin should be better suited to understand the complex competition and crosstalk of many different clones interacting with different epitopes on a natural complex protein antigen ^78, 79^. However, for such experiments, it is important to develop methods that will allow the specific analysis of epitope-specific interactions of different B-cell clones. Without an understanding of whether clonal interactions happen because of BCR binding of overlapping epitopes or whether clones develop with less competition because they do bind different epitopes, it is impossible to conclude whether clones with different affinities compete for the antigen ^80^.

Ultimately, it has been estimated that nearly 50% of newly produced auto-reactive B cells can avoid deletion and are induced into an anergic state in peripheral lymphoid tissues ^81, 82^. Thus, perhaps B cells in the periphery are not in the same basal state as we thought they should be. The surface expression level of membrane IgM is downregulated on anergic B cells ^83, 84^, indicating that their BCR signaling is somehow different from that of normal naïve B cells. Indeed, anergic B cells maintain a distinct gene expression profile ^81, 84^ and have been observed preferentially residing in T-zone areas ^85^, indicating that their surface chemokine receptor expression pattern is also different from that of naïve B cells, possibly because of their different signaling. Several studies have found that anergic B cells can be selected to become GC B cells, although these cells previously were thought to be non-responsive ^84, 86^. By recruiting anergic B cells into the GC response involving many rounds of mutation, these B cells may mutate away from their original auto-reactivity and become specific for antigens that may have a close relationship with autoantigens ^86– 89^. The mechanism—for example, whether anergic and normal B cells will follow the same rules of B-cell selection or which difference an anergic state can bring to B-cell activation and fate decisions at the T–B border—is still not clear.

## Concluding remarks

Co-stimulation from T cells plays indispensable roles in B-cell fate decisions after activation and has been studied in some detail. However, BCR signaling strength and patterns not only affect B-cell selection during development but also have been shown to affect the differentiation of B1, MZ, or Fo B-cell populations. Furthermore, BCR signaling strength may affect antigen-induced B-cell activation, migration, and surface co-stimulator molecule expression levels. It seems that there is still work to be done on how differential BCR signaling influences the differential development of B cells and how the various stages of antigen-induced T–B cell interactions affect further B-cell fate decisions.

## References

[1] Ueber das Zustandekommen der Diphtherie-Immunität und der Tetanus-Immunität bei Thieren. *Dtsch med Wochenschr.* 1890;16(49):1113–4. 10.1055/s-0029-1207589 5843503

[2] Fagraeus A: The plasma cellular reaction and its relation to the formation of antibodies *in vitro*. *J Immunol.* 1948;58(1):1–13. 18897986

[3] Cooper MD Peterson RD Good RA: Delineation of the Thymic and Bursal Lymphoid Systems in the Chicken. *Nature.* 1965;205:143–6. 10.1038/205143a0 14276257

[4] Cooper MD: The early history of B cells. *Nat Rev Immunol.* 2015;15(3):191–7. 10.1038/nri3801 25656707

[5] Vale AM Kearney JF Nobrega A: Development and Function of B Cell Subsets, Molecular Biology of B Cells.in Alt FW, *et al*Editors. Elsevier.2015;99–119. 10.1016/B978-0-12-397933-9.00007-2

[6] Hardy RR Hayakawa K: B cell development pathways. *Annu Rev Immunol.* 2001;19:595–621. 10.1146/annurev.immunol.19.1.595 11244048

[7] Kondo M Weissman IL Akashi K: Identification of clonogenic common lymphoid progenitors in mouse bone marrow. *Cell.* 1997;91(5):661–72. 10.1016/S0092-8674(00)80453-5 9393859

[8] Li YS Wasserman R Hayakawa K: Identification of the earliest B lineage stage in mouse bone marrow. *Immunity.* 1996;5(6):527–35. 10.1016/S1074-7613(00)80268-X 8986713

[9] Kraus M Alimzhanov MB Rajewsky N: Survival of resting mature B lymphocytes depends on BCR signaling via the Igalpha/beta heterodimer. *Cell.* 2004;117(6):787–800. 10.1016/j.cell.2004.05.014 15186779

[10] Lam KP Kühn R Rajewsky K: *In vivo* ablation of surface immunoglobulin on mature B cells by inducible gene targeting results in rapid cell death. *Cell.* 1997;90(6):1073–83. 10.1016/S0092-8674(00)80373-6 9323135

[11] Srinivasan L Sasaki Y Calado DP: PI3 kinase signals BCR-dependent mature B cell survival. *Cell.* 2009;139(3):573–86. 10.1016/j.cell.2009.08.041 19879843 PMC2787092

[12] Schweighoffer E Tybulewicz VL: Signalling for B cell survival. *Curr Opin Cell Biol.* 2017;51:8–14. 10.1016/j.ceb.2017.10.002 29149682

[13] Ichii M Oritani K Kanakura Y: Early B lymphocyte development: Similarities and differences in human and mouse. *World J Stem Cells.* 2014;6(4):421–31. 10.4252/wjsc.v6.i4.421 25258663 PMC4172670

[14] MacLennan IC Toellner KM Cunningham AF: Extrafollicular antibody responses. *Immunol Rev.* 2003;194:8–18. 10.1034/j.1600-065X.2003.00058.x 12846803

[15] MacLennan IC: Germinal centers. *Annu Rev Immunol.* 1994;12:117–39. 10.1146/annurev.iy.12.040194.001001 8011279

[16] Victora GD Nussenzweig MC: Germinal centers. *Annu Rev Immunol.* 2012;30:429–57. 10.1146/annurev-immunol-020711-075032 22224772

[17] Dal Porto JM Gauld SB Merrell KT: B cell antigen receptor signaling 101. *Mol Immunol.* 2004;41(6–7):599–613. 10.1016/j.molimm.2004.04.008 15219998

[18] Kurosaki T Shinohara H Baba Y: B cell signaling and fate decision. *Annu Rev Immunol.* 2010;28:21–55. 10.1146/annurev.immunol.021908.132541 19827951

[19] Packard TA Cambier JC: B lymphocyte antigen receptor signaling: initiation, amplification, and regulation. *F1000Prime Rep.* 2013;5:40. 10.12703/P5-40 24167721 PMC3790562

[20] Pierce SK Liu W: The tipping points in the initiation of B cell signalling: how small changes make big differences. *Nat Rev Immunol.* 2010;10(11):767–77. 10.1038/nri2853 20935671 PMC3406597

[21] Su TT Guo B Kawakami Y: PKC-beta controls I kappa B kinase lipid raft recruitment and activation in response to BCR signaling. *Nat Immunol.* 2002;3(8):780–6. 10.1038/ni823 12118249

[22] Avrameas S: Natural autoantibodies: from 'horror autotoxicus' to 'gnothi seauton'. *Immunol Today.* 1991;12(5):154–9. 10.1016/S0167-5699(05)80045-3 1715166

[23] Ochsenbein AF Fehr T Lutz C: Control of early viral and bacterial distribution and disease by natural antibodies. *Science.* 1999;286(5447):2156–9. 10.1126/science.286.5447.2156 10591647

[24] McKay JT Haro MA Daly CA: PD-L2 Regulates B-1 Cell Antibody Production against Phosphorylcholine through an IL-5-Dependent Mechanism. *J Immunol.* 2017;199(6):2020–9. 10.4049/jimmunol.1700555 28768724 PMC5587397

[25] Wang X Ma K Chen M: IL-17A Promotes Pulmonary B-1a Cell Differentiation via Induction of Blimp-1 Expression during Influenza Virus Infection. *PLoS Pathog.* 2016;12(1):e1005367. 10.1371/journal.ppat.1005367 26735852 PMC4703366

[26] Baumgarth N: Innate-like B cells and their rules of engagement. *Adv Exp Med Biol.* 2013;785:57–66. 10.1007/978-1-4614-6217-0_7 23456838

[27] Ghosn EE Sadate-Ngatchou P Yang Y: Distinct progenitors for B-1 and B-2 cells are present in adult mouse spleen. *Proc Natl Acad Sci U S A.* 2011;108(7):2879–84. 10.1073/pnas.1019764108 21282663 PMC3041118

[28] Savage HP Yenson VM Sawhney SS: Blimp-1-dependent and -independent natural antibody production by B-1 and B-1-derived plasma cells. *J Exp Med.* 2017;214(9):2777–94. 10.1084/jem.20161122 28698287 PMC5584113

[29] Cariappa A Takematsu H Liu H: B cell antigen receptor signal strength and peripheral B cell development are regulated by a 9-O-acetyl sialic acid esterase. *J Exp Med.* 2009;206(1):125–38. 10.1084/jem.20081399 19103880 PMC2626685

[30] Cariappa A Tang M Parng C: The follicular versus marginal zone B lymphocyte cell fate decision is regulated by Aiolos, Btk, and CD21. *Immunity.* 2001;14(5):603–15. 10.1016/S1074-7613(01)00135-2 11371362

[31] Gibb DR El Shikh M Kang DJ: ADAM10 is essential for Notch2-dependent marginal zone B cell development and CD23 cleavage *In vivo*. *J Exp Med.* 2010;207(3):623–35. 10.1084/jem.20091990 20156974 PMC2839139

[32] Hammad H Vanderkerken M Pouliot P: Transitional B cells commit to marginal zone B cell fate by Taok3-mediated surface expression of ADAM10. *Nat Immunol.* 2017;18(3):313–20. 10.1038/ni.3657 28068307

[33] Nguyen TT Kläsener K Zürn C: The IgM receptor FcμR limits tonic BCR signaling by regulating expression of the IgM BCR. *Nat Immunol.* 2017;18(3):321–33. 10.1038/ni.3677 28135254 PMC5310993

[34] Pao LI Lam KP Henderson JM: B cell-specific deletion of protein-tyrosine phosphatase Shp1 promotes B-1a cell development and causes systemic autoimmunity. *Immunity.* 2007;27(1):35–48. 10.1016/j.immuni.2007.04.016 17600736

[35] Tanigaki K Han H Yamamoto N: Notch-RBP-J signaling is involved in cell fate determination of marginal zone B cells. *Nat Immunol.* 2002;3(5):443–50. 10.1038/ni793 11967543

[36] Tsiantoulas D Kiss M Bartolini-Gritti B: Secreted IgM deficiency leads to increased BCR signaling that results in abnormal splenic B cell development. *Sci Rep.* 2017;7(1): 3540. 10.1038/s41598-017-03688-8 28615655 PMC5471202

[37] Matthews RJ Bowne DB Flores E: Characterization of hematopoietic intracellular protein tyrosine phosphatases: description of a phosphatase containing an SH2 domain and another enriched in proline-, glutamic acid-, serine-, and threonine-rich sequences. *Mol Cell Biol.* 1992;12(5):2396–405. 10.1128/MCB.12.5.2396 1373816 PMC364412

[38] Plutzky J Neel BG Rosenberg RD: Isolation of a src homology 2-containing tyrosine phosphatase. *Proc Natl Acad Sci U S A.* 1992;89(3):1123–7. 10.1073/pnas.89.3.1123 1736296 PMC48398

[39] Yi TL Cleveland JL Ihle JN: Protein tyrosine phosphatase containing SH2 domains: characterization, preferential expression in hematopoietic cells, and localization to human chromosome 12p12-p13. *Mol Cell Biol.* 1992;12(2):836–46. 10.1128/MCB.12.2.836 1732748 PMC364317

[40] Bannish G Fuentes-Pananá EM Cambier JC: Ligand-independent signaling functions for the B lymphocyte antigen receptor and their role in positive selection during B lymphopoiesis. *J Exp Med.* 2001;194(11):1583–96. 10.1084/jem.194.11.1583 11733573 PMC2193524

[41] Mårtensson IL Ceredig R: Review article: role of the surrogate light chain and the pre-B-cell receptor in mouse B-cell development. *Immunology.* 2000;101(4):435–41. 10.1046/j.1365-2567.2000.00151.x 11122446 PMC2327112

[42] Waisman A Kraus M Seagal J: IgG1 B cell receptor signaling is inhibited by CD22 and promotes the development of B cells whose survival is less dependent on Ig alpha/beta. *J Exp Med.* 2007;204(4):747–58. 10.1084/jem.20062024 17420268 PMC2118546

[43] Horikawa K Martin SW Pogue SL: Enhancement and suppression of signaling by the conserved tail of IgG memory-type B cell antigen receptors. *J Exp Med.* 2007;204(4):759–69. 10.1084/jem.20061923 17420266 PMC2118534

[44] Niiro H Clark EA: Regulation of B-cell fate by antigen-receptor signals. *Nat Rev Immunol.* 2002;2(12):945–56. 10.1038/nri955 12461567

[45] Paus D Phan TG Chan TD: Antigen recognition strength regulates the choice between extrafollicular plasma cell and germinal center B cell differentiation. *J Exp Med.* 2006;203(4):1081–91. 10.1084/jem.20060087 16606676 PMC2118299

[46] Schwickert TA Victora GD Fooksman DR: A dynamic T cell-limited checkpoint regulates affinity-dependent B cell entry into the germinal center. *J Exp Med.* 2011;208(6):1243–52. 10.1084/jem.20102477 21576382 PMC3173244

[47] Victora GD Schwickert TA Fooksman DR: Germinal center dynamics revealed by multiphoton microscopy with a photoactivatable fluorescent reporter. *Cell.* 2010;143(4):592–605. 10.1016/j.cell.2010.10.032 21074050 PMC3035939

[48] Pillai S Cariappa A Moran ST: Marginal zone B cells. *Annu Rev Immunol.* 2005;23:161–96. 10.1146/annurev.immunol.23.021704.115728 15771569

[49] Ansel KM Ngo VN Hyman PL: A chemokine-driven positive feedback loop organizes lymphoid follicles. *Nature.* 2000;406(6793):309–14. 10.1038/35018581 10917533

[50] Förster R Mattis AE Kremmer E: A putative chemokine receptor, BLR1, directs B cell migration to defined lymphoid organs and specific anatomic compartments of the spleen. *Cell.* 1996;87(6):1037–47. 10.1016/S0092-8674(00)81798-5 8978608

[51] Reif K Ekland EH Ohl L: Balanced responsiveness to chemoattractants from adjacent zones determines B-cell position. *Nature.* 2002;416(6876):94–9. 10.1038/416094a 11882900

[52] Arnon TI Horton RM Grigorova IL: Visualization of splenic marginal zone B-cell shuttling and follicular B-cell egress. *Nature.* 2013;493(7434):684–8. 10.1038/nature11738 23263181 PMC3561487

[53] Cinamon G Zachariah MA Lam OM: Follicular shuttling of marginal zone B cells facilitates antigen transport. *Nat Immunol.* 2008;9(1):54–62. 10.1038/ni1542 18037889 PMC2488964

[54] Gray D MacLennan IC Bazin H: Migrant mu+ delta+ and static mu+ delta- B lymphocyte subsets. *Eur J Immunol.* 1982;12(7):564–9. 10.1002/eji.1830120707 6811288

[55] Gray D Kumararatne DS Lortan J: Relation of intra-splenic migration of marginal zone B cells to antigen localization on follicular dendritic cells. *Immunology.* 1984;52(4):659–69. 6378770 PMC1454654

[56] Oliver AM Martin F Gartland GL: Marginal zone B cells exhibit unique activation, proliferative and immunoglobulin secretory responses. *Eur J Immunol.* 1997;27(9):2366–74. 10.1002/eji.1830270935 9341782

[57] Vinuesa CG Sze DM Cook MC: Recirculating and germinal center B cells differentiate into cells responsive to polysaccharide antigens. *Eur J Immunol.* 2003;33(2):297–305. 10.1002/immu.200310003 12548560

[58] Loder F Mutschler B Ray RJ: B cell development in the spleen takes place in discrete steps and is determined by the quality of B cell receptor-derived signals. *J Exp Med.* 1999;190(1):75–89. 10.1084/jem.190.1.75 10429672 PMC2195560

[59] Tan JB Xu K Cretegny K: Lunatic and manic fringe cooperatively enhance marginal zone B cell precursor competition for delta-like 1 in splenic endothelial niches. *Immunity.* 2009;30(2):254–63. 10.1016/j.immuni.2008.12.016 19217325

[60] Sáez de Guinoa J Barrio L Mellado M: CXCL13/CXCR5 signaling enhances BCR-triggered B-cell activation by shaping cell dynamics. *Blood.* 2011;118(6):1560–9. 10.1182/blood-2011-01-332106 21659539

[61] Calpe E Codony C Baptista MJ: ZAP-70 enhances migration of malignant B lymphocytes toward CCL21 by inducing CCR7 expression via IgM-ERK1/2 activation. *Blood.* 2011;118(16):4401–10. 10.1182/blood-2011-01-333682 21865343

[62] Okada T Cyster JG: B cell migration and interactions in the early phase of antibody responses. *Curr Opin Immunol.* 2006;18(3):278–85. 10.1016/j.coi.2006.02.005 16516453

[63] Gatto D Paus D Basten A: Guidance of B cells by the orphan G protein-coupled receptor EBI2 shapes humoral immune responses. *Immunity.* 2009;31(2):259–69. 10.1016/j.immuni.2009.06.016 19615922

[64] Pereira JP Kelly LM Xu Y: EBI2 mediates B cell segregation between the outer and centre follicle. *Nature.* 2009;460(7259):1122–6. 10.1038/nature08226 19597478 PMC2809436

[65] Yuseff MI Pierobon P Reversat A: How B cells capture, process and present antigens: a crucial role for cell polarity. *Nat Rev Immunol.* 2013;13(7):475–86. 10.1038/nri3469 23797063

[66] Allen RC Armitage RJ Conley ME: CD40 ligand gene defects responsible for X-linked hyper-IgM syndrome. *Science.* 1993;259(5097):990–3. 10.1126/science.7679801 7679801

[67] Ferrari S Giliani S Insalaco A: Mutations of *CD40* gene cause an autosomal recessive form of immunodeficiency with hyper IgM. *Proc Natl Acad Sci U S A.* 2001;98(22):12614–9. 10.1073/pnas.221456898 11675497 PMC60102

[68] Han S Hathcock K Zheng B: Cellular interaction in germinal centers. Roles of CD40 ligand and B7-2 in established germinal centers. *J Immunol.* 1995;155(2):556–67. 7541819

[69] Dal Porto JM Haberman AM Kelsoe G: Very low affinity B cells form germinal centers, become memory B cells, and participate in secondary immune responses when higher affinity competition is reduced. *J Exp Med.* 2002;195(9):1215–21. 10.1084/jem.20011550 11994427 PMC2193705

[70] Batista FD Neuberger MS: B cells extract and present immobilized antigen: implications for affinity discrimination. *EMBO J.* 2000;19(4):513–20. 10.1093/emboj/19.4.513 10675320 PMC305589

[71] Zaretsky I Atrakchi O Mazor RD: ICAMs support B cell interactions with T follicular helper cells and promote clonal selection. *J Exp Med.* 2017;214(11):3435–48. 10.1084/jem.20171129 28939548 PMC5679169

[72] Khalil AM Cambier JC Shlomchik MJ: B cell receptor signal transduction in the GC is short-circuited by high phosphatase activity. *Science.* 2012;336(6085):1178–81. 10.1126/science.1213368 22555432 PMC3777391

[73] Luo W Weisel F Shlomchik MJ: B Cell Receptor and CD40 Signaling Are Rewired for Synergistic Induction of the c-Myc Transcription Factor in Germinal Center B Cells. *Immunity.* 2018;48(2):313–326.e5. 10.1016/j.immuni.2018.01.008 29396161 PMC5821563

[74] Nowosad CR Spillane KM Tolar P: Germinal center B cells recognize antigen through a specialized immune synapse architecture. *Nat Immunol.* 2016;17(7):870–7. 10.1038/ni.3458 27183103 PMC4943528

[75] Zhang Y Garcia-Ibanez L Toellner KM: Regulation of germinal center B-cell differentiation. *Immunol Rev.* 2016;270(1):8–19. 10.1111/imr.12396 26864101 PMC4755139

[76] Zhang Y Meyer-Hermann M George LA: Germinal center B cells govern their own fate via antibody feedback. *J Exp Med.* 2013;210(3):457–64. 10.1084/jem.20120150 23420879 PMC3600904

[77] Phan TG Amesbury M Gardam S: B cell receptor-independent stimuli trigger immunoglobulin (Ig) class switch recombination and production of IgG autoantibodies by anergic self-reactive B cells. *J Exp Med.* 2003;197(7):845–60. 10.1084/jem.20022144 12668643 PMC2193892

[78] Kuraoka M Schmidt AG Nojima T: Complex Antigens Drive Permissive Clonal Selection in Germinal Centers. *Immunity.* 2016;44(3):542–52. 10.1016/j.immuni.2016.02.010 26948373 PMC4794380

[79] Tas JM Mesin L Pasqual G: Visualizing antibody affinity maturation in germinal centers. *Science.* 2016;351(6277):1048–54. 10.1126/science.aad3439 26912368 PMC4938154

[80] Toellner KM Sze DM Zhang Y: What Are the Primary Limitations in B-Cell Affinity Maturation, and How Much Affinity Maturation Can We Drive with Vaccination? A Role for Antibody Feedback. *Cold Spring Harb Perspect Biol.* 2017; pii: a028795. 10.1101/cshperspect.a028795 28630078 PMC5932584

[81] Gauld SB Benschop RJ Merrell KT: Maintenance of B cell anergy requires constant antigen receptor occupancy and signaling. *Nat Immunol.* 2005;6(11):1160–7. 10.1038/ni1256 16200069

[82] Grandien A Fucs R Nobrega A: Negative selection of multireactive B cell clones in normal adult mice. *Eur J Immunol.* 1994;24(6):1345–52. 10.1002/eji.1830240616 8206094

[83] Quách TD Manjarrez-Orduño N Adlowitz DG: Anergic responses characterize a large fraction of human autoreactive naive B cells expressing low levels of surface IgM. *J Immunol.* 2011;186(8):4640–8. 10.4049/jimmunol.1001946 21398610 PMC3095097

[84] Sabouri Z Perotti S Spierings E: IgD attenuates the IgM-induced anergy response in transitional and mature B cells. *Nat Commun.* 2016;7:13381. 10.1038/ncomms13381 27830696 PMC5109548

[85] Cyster JG Hartley SB Goodnow CC: Competition for follicular niches excludes self-reactive cells from the recirculating B-cell repertoire. *Nature.* 1994;371(6496):389–95. 10.1038/371389a0 7522305

[86] Cappione A 3rd Anolik JH Pugh-Bernard A: Germinal center exclusion of autoreactive B cells is defective in human systemic lupus erythematosus. *J Clin Invest.* 2005;115(11):3205–16. 10.1172/JCI24179 16211091 PMC1242189

[87] Reed JH Jackson J Christ D: Clonal redemption of autoantibodies by somatic hypermutation away from self-reactivity during human immunization. *J Exp Med.* 2016;213(7):1255–65. 10.1084/jem.20151978 27298445 PMC4925023

[88] Sabouri Z Schofield P Horikawa K: Redemption of autoantibodies on anergic B cells by variable-region glycosylation and mutation away from self-reactivity. *Proc Natl Acad Sci U S A.* 2014;111(25):E2567–75. 10.1073/pnas.1406974111 24821781 PMC4078846

[89] Williams JM Bonami RH Hulbert C: Reversing Tolerance in Isotype Switch-Competent Anti-Insulin B Lymphocytes. *J Immunol.* 2015;195(3):853–64. 10.4049/jimmunol.1403114 26109644 PMC4506889

